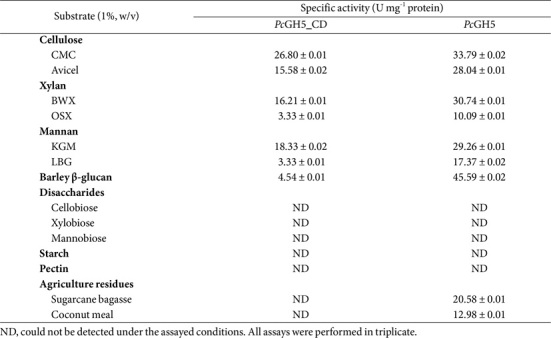# Non-Catalytic Domains of Glycoside Hydrolase Family 5 from *Paenibacillus curdlanolyticus* are Important for Promoting Multifunctional Enzyme Activities and Degradation of Agricultural Residues

**DOI:** 10.4014/jmb.2501.01046

**Published:** 2025-05-15

**Authors:** Niendy Virnanda Fatmawati, Apinya Singkhala, Prattana Ketbot, Sirilak Baramee, Rattiya Waeonukul, Chakrit Tachaapaikoon, Ayaka Uke, Akihiko Kosugi, Khanok Ratanakhanokchai, Patthra Pason

**Affiliations:** 1 Division of Biochemical Technology, School of Bioresources and Technology, King Mongkut's University of Technology Thonburi, Bangkok 10150, Thailand; 2 Excellent Center of Enzyme Technology and Microbial Utilization, Pilot Plant Development and Training Institute, King Mongkut's University of Technology Thonburi, Bangkok 10150, Thailand; 3 Biological Resources and Post-harvest Division, Japan International Research Center for Agricultural Sciences, Tsukuba, Ibaraki 305-8686, Japan

**Keywords:** Agricultural residue, glycoside hydrolase family 5, multifunctional enzyme, non-catalytic domain, oligosaccharide, *Paenibacillus curdlanolyticus*

## Abstract

*Pc*GH5 from *Paenibacillus curdlanolyticus* strain B-6 is a modular protein consisting of a catalytic domain of glycoside hydrolase family 5 (GH5), and three non-catalytic domains (a family 11 carbohydrate-binding module (CBM11), a fibronectin type 3 (Fn3), and a family 3 carbohydrate-binding module (CBM3). In this study, the recombinants full-length *Pc*GH5 and the catalytic domain (*Pc*GH5_CD) were expressed in *Escherichia coli* and purified. Most GH5 members exhibit endo-cellulase activity. However, the catalytic domain enzyme of strain B-6 exhibited unique properties, showing multifunctional enzyme activities of endo-cellulase, endo-xylanase, endo-mannanase, and endo-1,3-1,4-β-glucanase. The sequence alignment of *Pc*GH5_CD compared to other characterized GH5 enzymes suggests that the two catalytic residues and the six substrate-binding subsites of endo-cellulases were conserved with other different GH5 enzyme properties. Whereas a few conserved amino acid residues and/or short peptides located outside the active site of the GH5 endo-cellulases may be involved in broad substrate specificity of *Pc*GH5_CD enzyme on xylan, mannan and 1,3-1,4-β-glucan. Moreover, the non-catalytic domains (CBM11-Fn3-CBM3) linked to the GH5 catalytic domain are important for promoting the multifunctional enzyme activities of *Pc*GH5 on the β-1,4 glycosidic linkages of crystalline cellulose, highly branched polysaccharides, and β-1,4-1,6 and β-1,3-1,4 glycosidic linkages of polysaccharides, especially for the polysaccharides complex contained in agricultural residues. The full-length *Pc*GH5 is effective in producing oligosaccharides from agricultural residues without pretreatment. Therefore, it is interesting to use it as a source of prebiotics producer for use in various food products.

## Introduction

Agricultural residues are attractive raw materials because they are non-edible plant components, abundant, low cost and considered suitable feedstocks for producing value-added compounds. They consist of three main components: cellulose, hemicellulose and lignin. Cellulose and hemicellulose are major components of agricultural residues due to their potential as raw materials for the production of oligosaccharides [1]. Cellulose is the most abundant carbon source in nature, accounting for approximately 35%–50% of a plant’s dry weight, where cellulolytic enzymes are important for hydrolysing cellulose [2]. Hemicellulose constitutes approximately 25%–35% of plant dry weight and represents the second most abundant renewable biomass in nature [3]. Xylan and mannan are the two hemicelluloses most commonly found in agricultural residues. The main enzymes in the degradation of these two hemicelluloses are xylanolytic and mannanolytic enzymes, respectively [1].

Among cellulolytic and hemicellulolyic enzymes, endo-cellulases and endo-hemicellulases, which randomly hydrolyse the β-1,4 glycosidic linkages of cellulose and hemicelluloses to generate oligosaccharides, are the most efficient enzymes for degrading polysaccharides present in agricultural residues [4]. Among these, cello-oligosaccharides, manno-oligosaccharides and xylo-oligosaccharides can be used for a variety of purposes, especially as prebiotics in food products [1]. Alternatively, β-1,3-1,4-glucan is abundant in cereal grains, while endo-β-1,3-1,4-glucanases are vital for hydrolysing glucan into gluco-oligosaccharides, which can be used as prebiotics [5].

In general, the production of oligosaccharides from polysaccharides contained in agricultural residues by enzymatic hydrolysis requires pretreatment processes to reduce the complexity of plant biomass structure. However, pretreatment, for example, chemical pretreatment, is one of the most expensive steps in sugar production from agricultural residues, which increases the cost of sugar production and water treatment processes. Furthermore, the formation of aromatic aldehydes during chemical pretreatment prevents sugar formation by inhibiting enzyme activities [6]. Therefore, producing oligosaccharides from agricultural residues without chemical pretreatment using environmentally friendly enzymes is desirable.

Based on sequence homologies, 189 families of glycoside hydrolases (GHs) have been identified from a variety of microorganisms (http://www.cazy.org/Glycoside-Hydrolases.html). However, only a few of these GH families, such as a group of family 5 GHs, are representative of multifunctional enzymes [7]. Multifunctional enzymes, which are single protein products comprising several glycoside hydrolase activities, tend to be less complex and less expensive to manufacture compared to combinations of several different GH enzymes in terms of enzyme purification and enzyme loading [8]. Therefore, it would be interesting to study the multifunctional enzymes of the GH5 family.

The GH5 is one of the largest families of glycoside hydrolases, most of which are endo-cellulases, while other enzymes in this family are monofunctional enzymes (*e.g.* endo-mannanase, endo-xylanase, endo-β-1,3-1,4-glucanase, β-mannosidase, β-glucosidase, β-xylosidase); bifunctional enzymes (*e.g.* endo-cellulase/endo-mannanase, endo-cellulase/endo-xylanase, endo-cellulase/endo-β-glucanase); and multifunctional enzymes (*e.g.* endo-cellulase/endo-mannanase/endo-xylanase, endo-cellulase/endo-mannanase/endo-β-glucanase) (https://www.cazy.org/GH5_characterized.html#pagination_FUNC). Based on the similarity of amino acid sequence and biochemical characteristics, GH5 enzymes are currently divided into 55 subfamilies.

Many GH families consist of catalytic and ancillary non-catalytic modules, such as the carbohydrate-binding modules (CBMs) and the fibronectin type-3 homology (Fn3) module [9]. CBMs are classified into many families based on amino acid sequence similarities and functions. Generally, the function of CBMs is to support the enzymatic activity of the catalytic domain by promoting close contact between the substrate and the enzyme and supporting the catalytic module for easy digestion of the substrate [10]. Whereas Fn3 acts to modify the cellulose surface to increase the catalytic activity of cellulase against insoluble cellulose [11].

*Paenibacillus curdlanolyticus* strain B-6, a cellulolytic/xylanolytic/mannanolytic bacterium, has great potential in agricultural residue degradation [12]. We found one open reading frame carrying the *pcgh5* gene encoding a modular protein, including a catalytic domain belonging to GH5, and three non-catalytic modules; a family 11 carbohydrate-binding module (CBM11), a domain of fibronectin type 3 (Fn3) and a family 3 carbohydrate-binding module (CBM3). Compared with other characterized GH5 members, the GH5 catalytic domain of strain B-6 showed a low percent identity (38.0%–60.8%). Moreover, there are numerous non-catalytic modules associated with the GH5 catalytic domain of strain B-6. Thus, we expect to find new enzymatic properties of the *Pc*GH5 enzyme, while the three non-catalytic modules of *Pc*GH5 may play some role in *Pc*GH5 enzymatic activity.

In this study, *pcgh5* (GH5 catalytic domain linked with three non-catalytic domains) and *pcgh5_CD* (only GH5 catalytic domain) genes from *P. curdlanolyticus* strain B-6 were expressed in *Escherichia coli*, and the recombinant proteins were purified and characterized. Thereafter, the properties of *Pc*GH5 and the functional role of the non-catalytic domains in *Pc*GH5 were examined.

## Materials and Methods

### Chemicals

Carboxymethyl cellulose (CMC) (400–800 cps), Avicel (PH-101), glucose, cellobiose, birchwood xylan (BWX), oat spelt xylan (OSX), locust bean gum (LBG), barley β-glucan, rice starch, pectin and bovine serum albumin were purchased from Sigma-Aldrich (USA). Konjac glucomannan (KGM), cello-oligosaccharides (G2–G6), xylo-oligosaccharides (X2–X6) and manno-oligosaccharides (M2–M6) were purchased from Megazyme (Ireland). Mannose and xylose were purchased from Merck (KGaA, Germany). Other chemicals and reagents were of analytical grade.

### Organisms, Media and Growth Conditions

Chromosomal DNA was obtained from *P. curdlanolyticus* B-6, growing on Berg’s mineral salt medium at pH 7.0 [12]. Plasmid pET-28a(+) (Novagen, Germany), *E. coli* DH5α and *E. coli* BL21 (DE3) (New England Biolabs, USA) were used as the vector, cloning host and expression host, respectively. Luria-Bertani (LB) medium with an antibiotic (50 μg/ml kanamycin) was used to cultivate *E. coli* at 37°C to select *E. coli* transformants.

### Gene Cloning

*P. curdlanolyticus* B-6 was deposited in the BIOTEC Culture Collection of the National Center for Genetic Engineering and Biotechnology (BIOTEC), Thailand, under accession number BCC no. 11175. The chromosomal DNA of *P. curdlanolyticus* B-6 was extracted using phenol and chloroform. Oligonucleotide primers were constructed from the putative GH5 enzyme found in the partial genomic DNA sequence of the B-6 strain to clone the modular protein consisting of a catalytic domain of GH5 and three non-catalytic domains (*Pc*GH5), and its truncated protein contained only the catalytic GH5 domain (*Pc*GH5_CD). The primer containing *Bam*H1-HF and *Not*1-HF recognition sites (5'-GGGGG**GGATCC**GCTGAAGCTGTCGACTTAGAAAATCTG-3' was used for the *Pc*GH5 and *Pc*GH5_CD-forward primer, while 5'-TTTTT**GCGGCCGC**TTATGAAGGCTCTACGCCCC-3' for *Pc*GH5-reverse primer, and 5'-TTTTT**GCGGCCGC**TTATGAAGGCTCTACGCCCC-3' for *Pc*GH5_CD-reverse primer (*Bam*H1-HF and *Not*1-HF recognition sites are in bold) were used to amplify *Pc*GH5 and *Pc*GH5_CD fragments by PCR using B-6 strain genomic DNA as a template. Following the manufacturer’s instructions, PCR was carried out using Ex Taq DNA polymerases (New England Biolabs Ltd., UK). The DNA fragment was subjected to digestion using *Bam*H1-HF and *Not*1-HF (New England Biolabs, Ltd.), followed by an agarose gel run and extraction using a Qiagen, USA, Polymerase Chain Reaction (PCR) kit. After that, each resultant product was ligated between the pET28a vector’s *Bam*H1-HF and *Not*1-HF sites to create the pET28a*Pc*GH5 and pET28a*Pc*GH5_CD plasmids. After ligating the resultant products to create recombinant plasmids, the ligated combinations were transformed into *E. coli* DH5α. Positive clones were verified by using DNA sequencing and colony PCR.

### Sequence Alignment

The protein sequence of *Pc*GH5_CD was submitted to the NCBI database, which immediately collected the high-similarity proteins for further characterization (https://blast.ncbi.nlm.nih.gov/Blast.cgi). Moreover, these protein sequences were evaluated using the Clustal Omega multiple sequence alignment (https://www.ebi.ac.uk/jdispatcher/msa/clustalo).

### Subfamily of *Pc*GH5_CD Enzyme

The amino acid sequence of the *Pc*GH5_CD enzyme was analysed to identify its subfamily classification. BLAST analysis was performed using the NCBI protein database [13] to identify homologous sequences, prioritizing well-characterized GH5 enzymes. Multiple sequence alignment was conducted using the MUSCLE algorithm in MEGA11 software [14], with ClustalW on the EMBL-EBI server (https://www.ebi.ac.uk/Tools/msa/clustalw2/) used in parallel to verify the robustness of the alignment and ensure consistent tree topology. The phylogenetic tree was constructed using the maximum likelihood method with 2,000 bootstrap replicates to enhance statistical robustness. To refine subfamily classification, additional BLAST searches were performed against the CAZy database [13] to assess sequence identity with known GH5 subfamilies. The subfamily classification was inferred based on phylogenetic clustering with well-characterized GH5 enzymes. The resulting tree topology was examined for congruence with established GH5 subfamily relationships [15], providing insights into the evolutionary placement of *Pc*GH5_CD enzyme within the glycoside hydrolase superfamily.

### Three-Dimensional Structure of *Pc*GH5_CD Enzyme

Three-dimensional structure (3D structure) of *Pc*GH5_CD enzyme was performed using structural modeling analysis. Homologous proteins were first identified by searching the Protein Data Bank (PDB) database (http://www.rcsb.org/pdb/home/home.do). The crystal structure (PDB ID: 4U3A) of bifunctional endo-cellulase/endo-xylanase *Ct*Cel5E from *Clostridium thermocellum* ATCC 27405 [16], which showed high sequence identity with the *Pc*GH5_CD catalytic domain (48.43%), was selected as the reference protein. The 3D structure of the *Pc*GH5_CD enzyme was built based on the 4U3A protein model by the SWISS-MODEL (https://www.swissmodel.expasy.org), generating a catalytic domain model. Ligands relevant to *Pc*GH5_CD enzyme, xylotetraose (X4), mannotetraose (M4), and 1,3-1,4-β-glucotetraose (G4) for endo-xylanase, endo-mannanase, and endo-1,3-1,4-β-glucanase, respectively, which interact with the *Pc*GH5_CD at subsites −2 to +2 were obtained from PubChem (https://pubchem.ncbi.nlm.nih.gov/) and used for molecular docking analysis with PyRx software to predict binding interactions [17]. Structural visualization and analysis were performed using PyMOL (https://pymol.org/), focusing on catalytic and binding sites, domain organization, and surface-exposed residues. Distance measurements between interacting residues and ligands were determined in angstroms (Å) using PyMOL to evaluate binding interactions and substrate specificity [18]. Structural modeling studies were performed to infer potential enzymes, namely, endo-xylanase, endo-mannanase, and endo-1,3-1,4-β-glucanase activities, based on ligands interactions with key residues within the *Pc*GH5_CD subsite.

### Expression and Purification of Recombinants *Pc*GH5_CD and *Pc*GH5

The DNA fragments pET28a_*Pc*GH5 and pET28a_*Pc*GH5_CD were transformed into *E. coli* BL21 (DE3). Subsequently, for expression, the transformants were grown in LB medium supplemented with kanamycin (50 μg/ml). The cultures were incubated at 200 rpm and 37°C until their OD_600_ reached 0.6. Isopropyl β-D-1-thiogalactopyranoside was added to the cultures at a final concentration of 1 mM to promote protein production, and they were then incubated at 16°C for an additional 18 h. After incubations, the cells were harvested, washed, and sonicated and the cells debris were eliminated by centrifugation at 8,000 rpm for 30 min at 4°C. Cell extracts were purified using an affinity chromatography HisTrap HP column (GE Healthcare, Japan), which immediately estimated the purity of protein recombinants by sodium dodecyl sulphate-polyacrylamide gel electrophoresis (SDS-PAGE) [19].

### Protein Determination and Enzyme Assays of *Pc*GH5_CD and *Pc*GH5

The protein concentration was ascertained using the Bradford method [20], with bovine serum albumin as the standard. Reaction mixtures for enzyme assays consisted of the purified enzyme (1 μM) and 1% (w/v) of each substrate in 50 mM sodium acetate buffer (pH 6.0) at 50°C, which are the optimum conditions of the GH5 enzyme for hydrolysis of the substrates. The amount of reducing sugars released from each substrate (CMC, Avicel (PH-101), BWX, OSX, KGM, LBG, barley β-glucan, starch and pectin) was measured using the Somogyi-Nelson method [21]. The amount of enzyme that released one μmol of reducing sugar per minute under assay conditions was defined as one unit (U) of enzyme activity. For cellobiose, xylobiose or mannobiose, the hydrolysis product was analysed by high-performance liquid chromatography (HPLC) with a RID10A reflective index detector (Shimadzu RID-10A, Japan). The sample was separated on the BP-200 Ag carbohydrate column (Benson polymeric, USA) with a mobile phase of water (HPLC Grade, Fisher Scientific, UK) at 85°C and a flow rate of 0.3 ml/min.

### Degradation Product Analysis

Enzyme activities of *Pc*GH5_CD and *Pc*GH5 on 1% (w/v) CMC, Avicel, BWX, OSX, KGM, LBG and barley β-glucan were performed using 500 μl of reaction mixture in sodium acetate buffer (pH 6.0) containing purified *Pc*GH5 enzyme (1 μM) at 50°C for 30 min. Analysis of the hydrolysis products released from the polysaccharides was performed by thin-layer chromatography (TLC) on silica gel (60 F245) plates (Merck, Germany) with a mixture of acetic acid, *n*-butanol and water (1:2:1) as the solvent system. The sugar spots were detected by heating the plate at 100°C after spraying with a reagent consisting of 4 g of a-diphenylamine, 4 ml of aniline, 200 ml of acetone and 30 ml of 80% (v/v) phosphoric acid [6].

### Preparation and Saccharification of Agricultural Residues

Sugarcane bagasse was obtained from Suphanburi province, Thailand, whereas coconut meal (a by-product of coconut milk production) was obtained from a coconut milk shop at a fresh market in Nonthaburi province, Thailand). The agricultural residues were finely chopped with scissors, ground in a blender, and finally sieved through a 45-mesh screen (Endecotts Ltd., England). After washing several times with warm distilled water to remove impurities, the samples were oven-dried at 50°C until a constant weight and stored for later use.

The agricultural residues (1%, w/v), sugarcane bagasse and coconut meal were hydrolysed with 5 μM purified *Pc*GH5 enzyme in sodium acetate buffer (pH 6.0) at 50°C for 30 min, 1 h and 5 h. Analysis of the hydrolysis products released from the agricultural residues was performed by TLC.

## Results and Discussion

### Modular Structure of *Pc*GH5

**Fig. 1:**
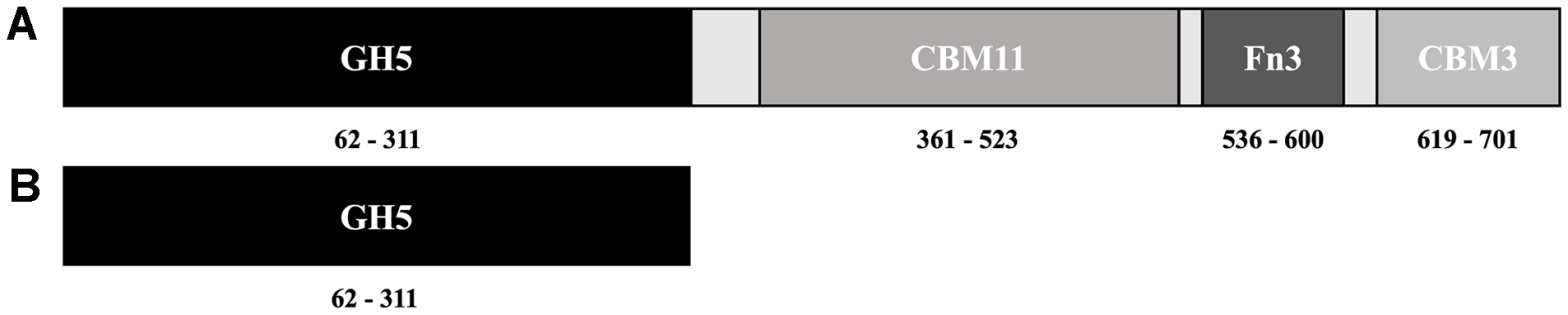
Schematic illustration of modular structures of (**A**) full-length *Pc*GH5 consisting of a GH5 catalytic domain, a family 11 carbohydrate-binding module (CBM11), a domain of fibronectin type 3 (Fn3), and a family 3 carbohydrate-binding module (CBM3) and (**B**) the GH5 catalytic domain (*Pc*GH5_CD) from *P. curdlanolyticus* B-6.

Based on the partial genome analysis of *P. curdlanolyticus* B-6, we identified the *pcgh5* gene, which consisted of 2,268 nucleotides and encoded the *Pc*GH5 protein comprising 756 amino acid residues with a predicted molecular mass of 85,176.94 Da (https://web.expasy.org/cgi-bin/compute_pi/pi_tool). A homology search using the NCBI database (https://www.ncbi.nlm.nih.gov/) demonstrated that *Pc*GH5 was a modular protein, consisting of a family GH5 catalytic domain and three non-catalytic domains: a CBM11, an Fn3 and a CBM3 sequentially from the *N*-terminus, extending from positions 62 to 311, 361 to 523, 536 to 600 and 619 to 701, respectively (Fig. 1A).

### GH5 Catalytic Domain from *P. curdlanolyticus* Strain B-6

**Fig. 2:**
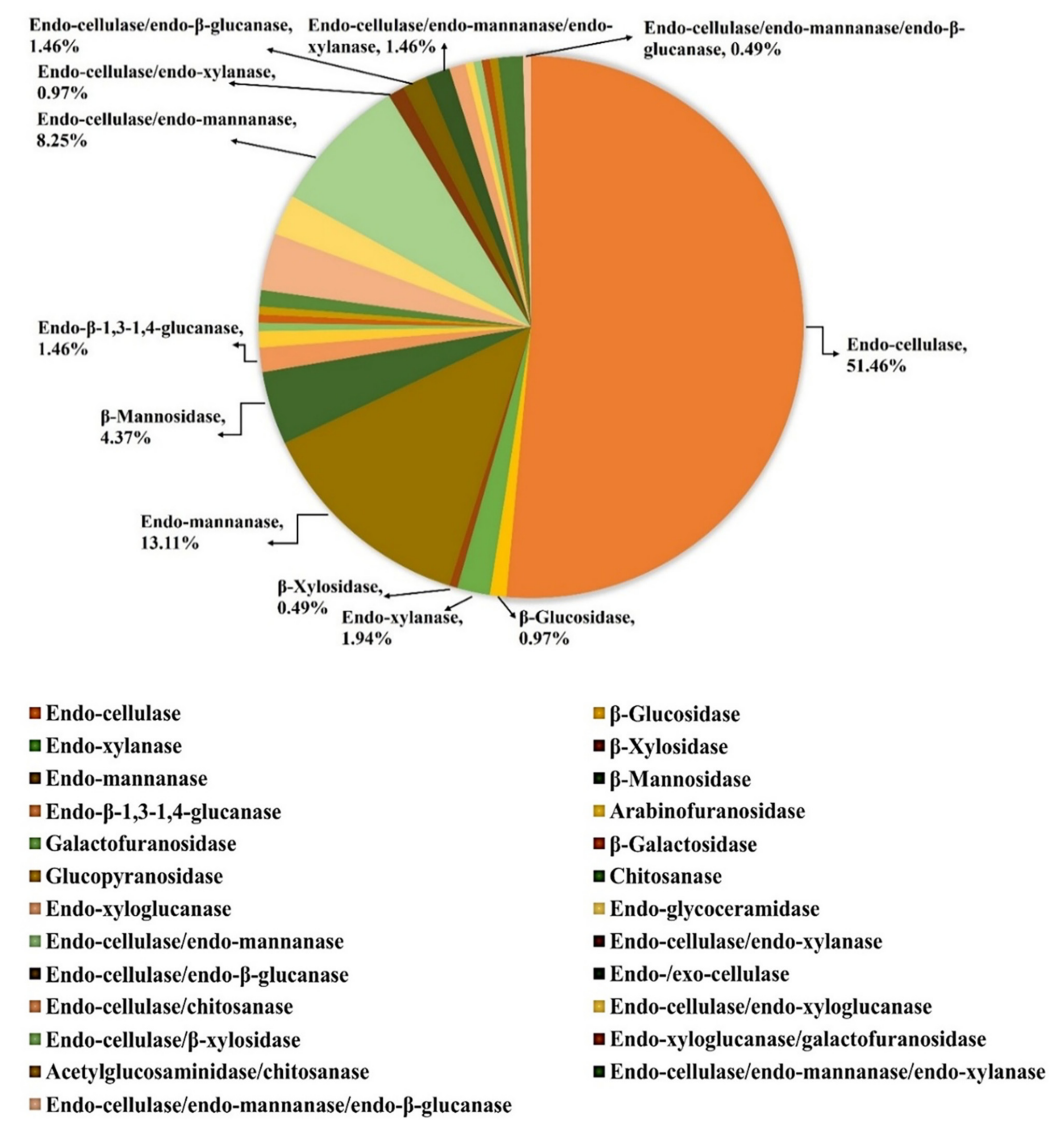
From the Carbohydrate-Active EnZymes database (CAZy) (https://www.cazy.org/GH5_characterized.html#pagination_FUNC), the graphic shows the percentage of different enzymes in the GH5 members with different enzymatic activities. Each color is coded according to the type of substrate and the specificity of the reaction.

The identified GH5 catalytic domain of strain B-6, designated as *Pc*GH5_CD (Fig. 1B) (GenBank ID: GFN32992.1), showed low identity (38.0%–60.8%) compared with other characterized GH5 members. According to the CAZy database (http://www.cazy.org/GH5.html), GH5 is one of the largest families of glycoside hydrolases, with 34,569 members reported in the GenBank database. However, only 638 members have been characterized, which are mainly endo-cellulases (Fig. 2). Analysis of the identity of the *Pc*GH5_CD enzyme (GH5 catalytic domain only) to other characterized GH5 enzymes with different properties in the GH5 family whose 3D structures have been elucidated indicates that the highest sequence identity was between the bifunctional endo-cellulase/endo-xylanase from *C. thermocellum* (48.43%) [16], endo-cellulase from *Thermoascus aurantiacus* (40.00%) [22] and bifunctional endo-cellulase/endo-mannanase from *Bacillus agaradhaerens* (38.68%) [23].

### Non-Catalytic Domains of *Pc*GH5 Enzyme from *P. curdlanolyticus* Strain B-6

The three non-catalytic domains (CBM11, CBM3 and Fn3) within the modular *Pc*GH5 protein from *P. curdlanolyticus* strain B-6 were examined. BlastP analysis of CBM11 in the *Pc*GH5 enzyme from strain B-6 with other characterized CBM11 members revealed sequence identities ranging from 25% to 47%. Among the characterized CBM11 proteins, the highest sequence similarity was observed with CBM11 from *Acetivibrio straminisolvens* (47.02%), followed by *C. thermocellum* AFN85452.1 (46.71%), *Acetivibrio thermocellus* (46.71%), *A. thermocellus* ATCC 27405 (46.47%) and *C. thermocellum* AFN85449.1 (46.2%).

BlastP analysis of the Fn3 non-catalytic domain in *Pc*GH5 from *P. curdlanolyticus* strain B-6 revealed sequence identities ranging from 48% to 50% with other characterized Fn3 members. The highest sequence similarities were found with the Fn3 domain from *Paenibacillus* sp. BIHB 4019 (50.00%), followed by *Paenibacillus* sp.(49.62%), *Paenibacillus xylanivorans* (49.62%), *Paenibacillus* sp. LS1 (48.85%) and *Paenibacillus* sp. FSL H8-0079 (48.85%).

According to the CAZy database (http://www.cazy.org/CBM3.html), only 22 proteins of characterized CBM3 modules have been reported. Using BlastP analysis, the CBM3 domain of *Pc*GH5 exhibited sequence identities between 46% and 60% compared to other characterized CBM3 members. The highest sequence similarity was observed with CBM3 from *Paenibacillus xylaniclasticus* (59.77%), followed by *Paenibacillus xylanexedens* (56.32%), *Paenibacillus terricola* (54.65%), *Paenibacillus cellulosilyticus* (53.49%) and *Paenibacillus* sp. CCS19 (51.14%).

### Subfamily of *Pc*GH5_CD Enzyme

**Fig. 3:**
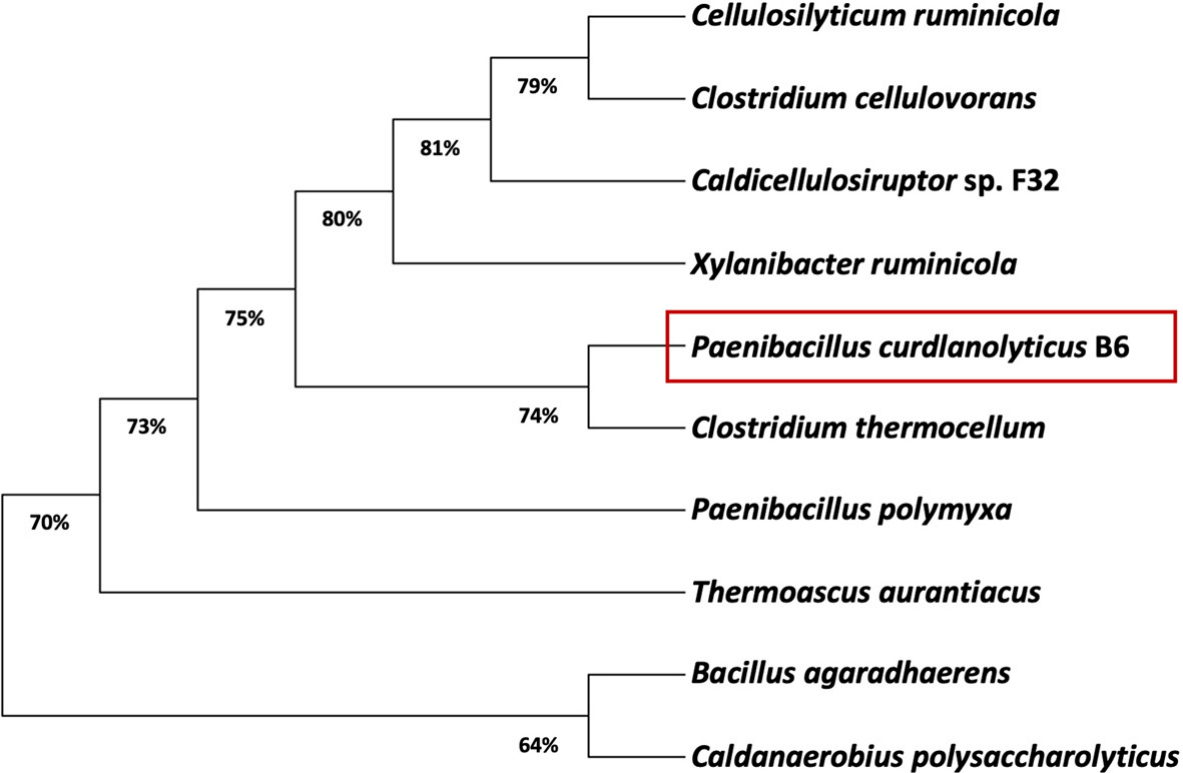
Phylogenetic tree of the *Pc*GH5_CD enzyme with other characterized members of GH5 subfamily based on amino acid sequences. The tree was constructed using MUSCLE in MEGA11 software [14] with 2,000 bootstrap replicates. The number for each node is the bootstrap percentage. Most members exhibited unknown subfamily, such as a bifunctional endo-cellulase/endo-xylanase from *C. thermocellum* (GenBank Accession No. 4U3A_1), an endo-β-1,3-1,4- glucanase from *P. polymyxa* (GenBank Accession No. QBY21421.1), a bifunctional endo-cellulase/endo-mannanase from *B. agaradhaerens* (GenBank Accession No. 2WHL_1), a multifunctional endo-cellulase/endo-mannanase/endo-β-1,3-1,4- glucanase from *C. polysaccharolyticus* (GenBank Accession No. AAD09354.1), and subfamily 4, such as an endo-β-1,3-1,4- glucanase from *Caldicellulosiruptor* sp. F32 (GenBank Accession No. AGM71677.1), a bifunctional endo-cellulase/endomannanase from *C. cellulovorans* (GenBank Accession No. AAD39739.1), a bifunctional endo-cellulase/endo-xylanase from *C. ruminicola* GenBank Accession No. ACZ98591.1), a bifunctional endo-cellulase/endo-xylanase from *X. ruminicola* (GenBank Accession No. AAC36862.1), while one member, endo-cellulase from *T. aurantiacus* (GenBank Accession No. AAL88714.2), exhibited subfamily 5.

The phylogenetic tree of *Pc*GH5_CD enzyme with other characterized GH5 members with high sequence identity (35.09–50.00%) was constructed by using the MUSCLE algorithm in MEGA11 software [14], with ClustalW on the EMBL-EBI server (https://www.ebi.ac.uk/Tools/msa/clustalw2/) used in parallel to verify the robustness of the alignment and ensure consistent tree topology. As shown in Fig. 3, *Pc*GH5_CD was individually separated from other GH5 members, including nine members of a bifunctional endo-cellulase/endo-xylanase from *C. thermocellum* (GenBank Accession No. 4U3A_1) (unknown subfamily), an endo-β-1,3-1,4-glucanase from *Paenibacillus polymyxa* (GenBank Accession No. QBY21421.1) (unknown subfamily), a bifunctional endo-cellulase/endo-mannanase from *B. agaradhaerens* (GenBank Accession No. 2WHL_1) (unknown subfamily), a multifunctional endo-cellulase/endo-mannanase/endo-β-1,3-1,4-glucanase from *Caldanaerobius polysaccharolyticus* (GenBank Accession No. AAD09354.1) (unknown subfamily), an endo-β-1,3-1,4-glucanase from *Caldicellulosiruptor* sp. F32 (GenBank Accession No. AGM71677.1) (subfamily 4), a bifunctional endo-cellulase/endo-mannanase from *Clostridium cellulovorans* (GenBank Accession No. AAD39739.1) (subfamily 4), a bifunctional endo-cellulase/endo-xylanase from *Cellulosilyticum ruminicola* (GenBank Accession No. ACZ98591.1) (subfamily 4), a bifunctional endo-cellulase/endo-xylanase from *Xylanibacter ruminicola* (GenBank Accession No. AAC36862.1)(subfamily 4), and an endo-cellulase from *T. aurantiacus* (GenBank Accession No. AAL88714.2) (subfamily 5). Although *Pc*GH5_CD is closely related to subfamilies 4 and 5, it cannot be placed in any of the known GH5 subfamilies and is therefore grouped with an unclassified enzyme. In 2012, Aspeborg *et al*. [15] classified GH5 enzymes into 51 subfamilies, while in 2022, Martins *et al*. [24] classified GH5 enzymes into 55 subfamilies, meaning that there are still many GH5 enzymes that need to be classified. Thus, phylogenetic analysis suggests that *Pc*GH5 may belong to an unidentified GH5 subfamily, which requires further studies to identify functional and evolutionary relationships within the GH5 family.

### Catalytic Site of *Pc*GH5_CD Enzyme

**Fig. 4:**
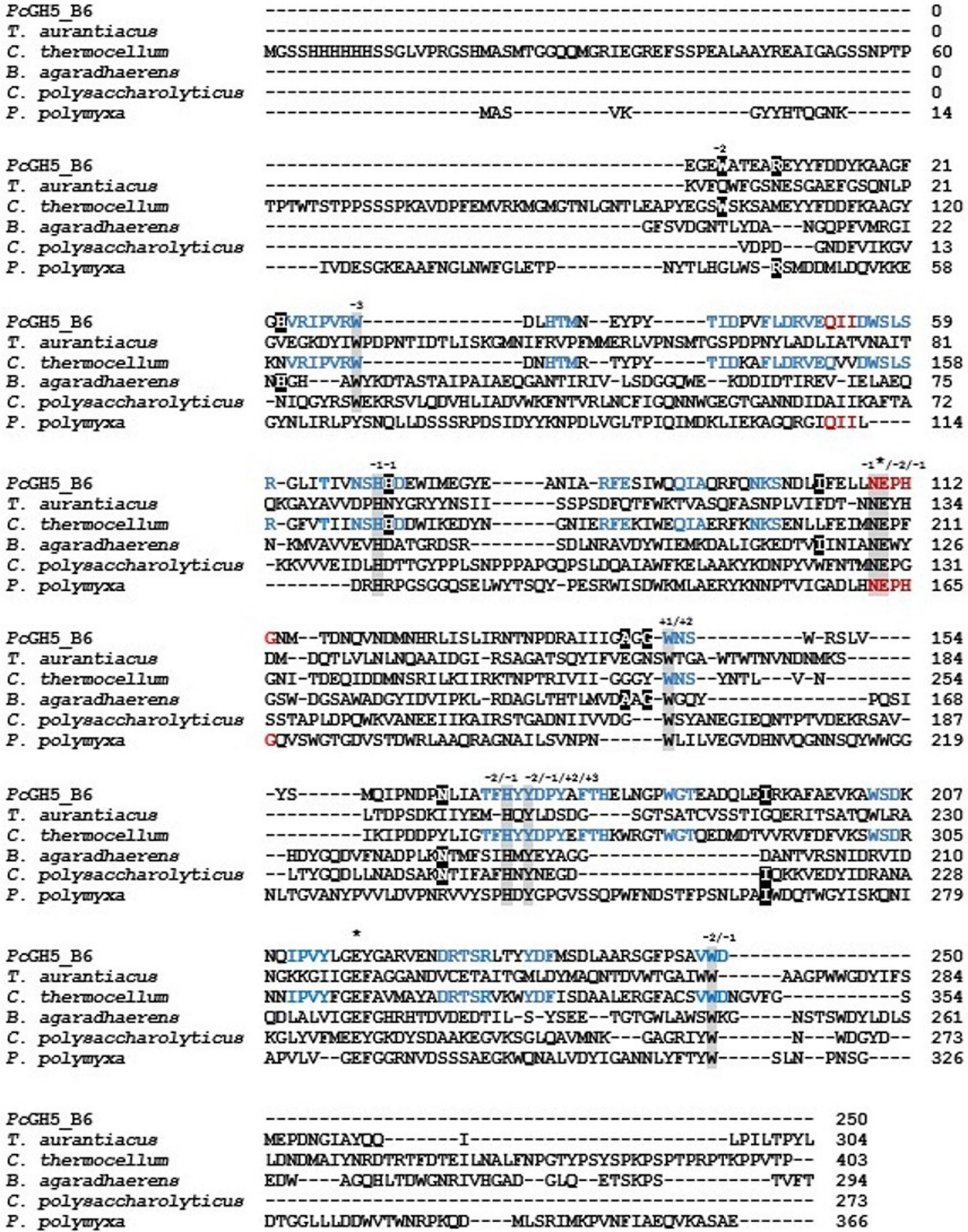
Amino acid sequence alignment of the catalytic domain enzyme from *P. curdlanolyticus* strain B-6 (this work) compared with other characterized GH5 enzymes with different functions such as endo-cellulase from *T. aurantiacus* (GenBank Accession No. AAL88714.2), bifunctional endo-cellulase/endo-xylanase from *C. thermocellum* (GenBank Accession No. 4U3A_1), bifunctional endo-cellulase/endo-mannanase from *B. agaradhaerens* (GenBank Accession No. 2WHL_1), multifunctional endo-cellulase/endo-mannanase/ endo-β-glucanase from *C. polysaccharolyticus* (GenBank Accession No. AAD09354.1), and endo-β-1,3-1,4- glucanase from *P. polymyxa* (GenBank Accession No. QBY21421.1). The two catalytic residues are marked by an asterisk, while the six substrate-binding subsites of endo-cellulase are highlighted in grey. The six substrate-binding subsites are numbered − 3, − 2, − 1, + 1, + 2 and + 3. Conserved residues of endo-xylanase, endo-mannanase, and endo-β-1,3-1,4-glucanase of GH5 members located outside the active site of endo-cellulase are highlighted in black. Blue letters represent conserved short peptides between the catalytic domain enzyme from *P. curdlanolyticus* strain B-6 and endo-xylanase, while red letters represent conserved short peptides between the catalytic domain enzyme from *P. curdlanolyticus* strain B-6 and β-1,3-1,4-glucanase. Alignment was constructed with the ClustalW program (https://www.ebi.ac.uk/jdispatcher/msa/clustalo).

The three-dimensional structures of some GH5 enzymes have been reported. To identify the conserved regions and consensus amino acids, the *Pc*GH5_CD sequence was subjected to multiple sequence alignments with other characterized GH5 enzyme properties using ClustalW on the EMBL-EBI server (https://www.ebi.ac.uk/Tools/msa/clustalw2/). Although, GH5 members having different enzyme activities and substrate preferences, such as endo-cellulase from *T. aurantiacus* [22], bifunctional endo-cellulase/endo-xylanase from *C. thermocellum* [16], bifunctional endo-cellulase/endo-mannanase from *B. agaradhaerens* [23], multifunctional endo-cellulase/endo-mannanase/endo-β-glucanase from *C. polysaccharolyticus* [7] and endo-β-1,3-1,4-glucanase from *P. polymyxa* KF-1 [25], two catalytic residues of endo-cellulase from *T. aurantiacus*, Glu132 (acid/base) and Glu239 (nucleophile) [22], were conserved in all types of characterized GH5 members, including *Pc*GH5_CD (Fig. 4).

### Substrate-Binding Subsites of *Pc*GH5_CD Enzyme

Most GH5 members exhibit endo-cellulase activity (Fig. 2). As shown in Table 1, the conserved 15 amino acid residues of the Cel5A endo-cellulase from *T. aurantiacus* [22], which are located in the six substrate-binding subsites within the active site, Trp30 at subsite −3; Glu132, His197, Tyr199 and Trp272 at subsite −2; His91, Asn130, Glu132, His197, Tyr199 and Trp272 at subsite −1; Trp169 at subsite +1; Trp169 and Tyr199 at subsite +2 and Tyr199 at subsite +3, were also conserved in almost all other GH5 enzyme properties, such as bifunctional endo-cellulase/endo-xylanase from *C. thermocellum* [16], bifunctional endo-cellulase/endo-mannanase from *B. agaradhaerens* [23], multifunctional endo-cellulase/endo-mannanase/endo-β-glucanase from *C. polysaccharolyticus* [7] and endo-β-1,3-1,4-glucanase from *P. polymyxa* KF-1 [25], as well as the GH5 catalytic domain from *P. curdlanolyticus* strain B-6.

Yuan, *et al*. [16] reported that conserved amino acid residues within the substrate-binding subsites of bifunctional endo-cellulase/endo-xylanase *Ct*Cel5E from *C. thermocellum*, namely His168, Asn208, Glu209, Tyr270 and Trp347 at subsite −1 and Trp347 at subsite −2, were important for not only cellulase but also xylanase activity. Moreover, compared with the endo-cellulase Cel5A from *T. aurantiacus*, some amino acid residues that are located outside but close to the active site of the GH5 endo-cellulases, such as Trp103 (subsite −2) and His169 (subsite −1) (see binding subsites of endo-cellulase Cel5A from *T. aurantiacus* in Table 1), which are critical for the endo-xylanase activity of the bifunctional endo-cellulase/endo-xylanase *Ct*Cel5E from *C. thermocellum* [16], were also found in Trp4 and His70 of the *Pc*GH5_CD enzyme. However, these two residues were not found in the other GH5 enzymes that lack xylanase activity, such as multifunctional endo-cellulase/endo-mannanase/endo-β-glucanase from *C. polysaccharolyticus*, bifunctional endo-cellulase/endo-mannanase from *B. agaradhaerens* and endo-β-1,3-1,4-glucanase PpBglu5A from *P. polymyxa* KF-1 (Table 1). On the other hand, when comparing the amino acid sequences between bifunctional endo-cellulase/endo-xylanase *Ct*Cel5E and the *Pc*GH5_CD enzyme, several other conserved amino acid residues, such as Thr64 and Asp250, and short peptides, such as 123VRIPVRW129, 132HTM134, 140TID142, 145FLDRVEQ151, 154DWSLSR159, 166NSHHD170, 183RFE185, 190QIA192, 197NKS199, 246WNS248, 266TFHYYDPY273, 275FTH277, 283WGT285, 302WSD304, 308IPVY311, 322DRTSR326, 330YDF332 and 346VWD348 (the numbers correspond to *Ct*Cel5E), were found (Fig. 4), some of which are predicted to be important for the xylanase activity of the *Pc*GH5_CD enzyme.

Two catalytic residues and all substrate-binding subsites from subsites –3 to +3 within the active site of the Cel5A endo-cellulase from *T. aurantiacus* [22] were conserved in the bifunctional endo-cellulase/endo-mannanase *Ba*Man5A from *B. agaradhaerens* [23] (Table 1). On the other hand, His24, Ile118, Ala158, Gly160 and Asn183 in the bifunctional cellulase/mannanase *Ba*Man5A were conserved with His23, Ile104, Ala144, Gly146 and Asn164 in *Pc*GH5_CD, respectively, but these residues were not found in endo-cellulase Cel5A from *T. aurantiacus*, bifunctional endo-cellulase/endo-xylanase *Ct*Cel5E from *C. thermocellum* and endo-β-1,3-1,4-glucanase from *P. polymyxa* KF-1 (Fig. 4). Therefore, some of these amino acids are likely related to the mannanase activity of *Pc*GH5_CD.

The β-1,3-1,4-glucanase PpBglu5A from *P. polymyxa* KF-1 [25] had low similarity with the *Pc*GH5_CD enzyme (35.09%). However, the conserved amino acid residues in the active site of β-1,3-1,4-glucanase PpBglu5A enzyme were similar to those of other GH5 enzymes, including the two catalytic residues and the substrate-binding subsites, except that this enzyme lacks a tryptophan at subsite −3 (Table 1). In fact, this position is weaker than the other subsites [23]. There are some amino acid residues, such as Arg9 and Ile194, and short peptides outside the active site that are conserved between PpBglu5A and *Pc*GH5_CD, namely 111QII113 and 162NEPHG166 (the numbers correspond to PpBglu5A), but these peptides are not found in other GH5 enzymes. These peptides may be involved in the β-1,3-1,4-glucanase activity of the *Pc*GH5_CD enzyme.

The results showed that due to the similarity in sugar structures (cyclic ring, –OH group, –H group), GH5 enzymes may be able to use amino acid residues within the substrate-binding subsites of the parent enzyme, endo-cellulase to bind to other structurally similar sugar substrates, such as the xylose-xylose linkage of xylan, the mannose-mannose, glucose-mannose and galactose-mannose linkage of mannans and the glucose-glucose linkage of β-glucan, together with a number of conserved amino acids and/or short peptides that are located outside but should be close to the active site of the GH5 endo-cellulases to bind and hydrolyse substrates. Moreover, these results support the evolution of GH5 endo-cellulases into endo-mannanases, endo-xylanases and endo-1,3-1,4-β-glucanases, using amino acid residues located within the six substrate-binding subsites of the endo-cellulase together with some mutant amino acids located near the active site to bind to other substrates (mannan, xylan, β-glucan) and then catalyse substrates by the two catalytic residues (Glu/Glu) of the cellulase.

### Structure Modeling of the *Pc*GH5_CD Enzyme

**Fig. 5:**
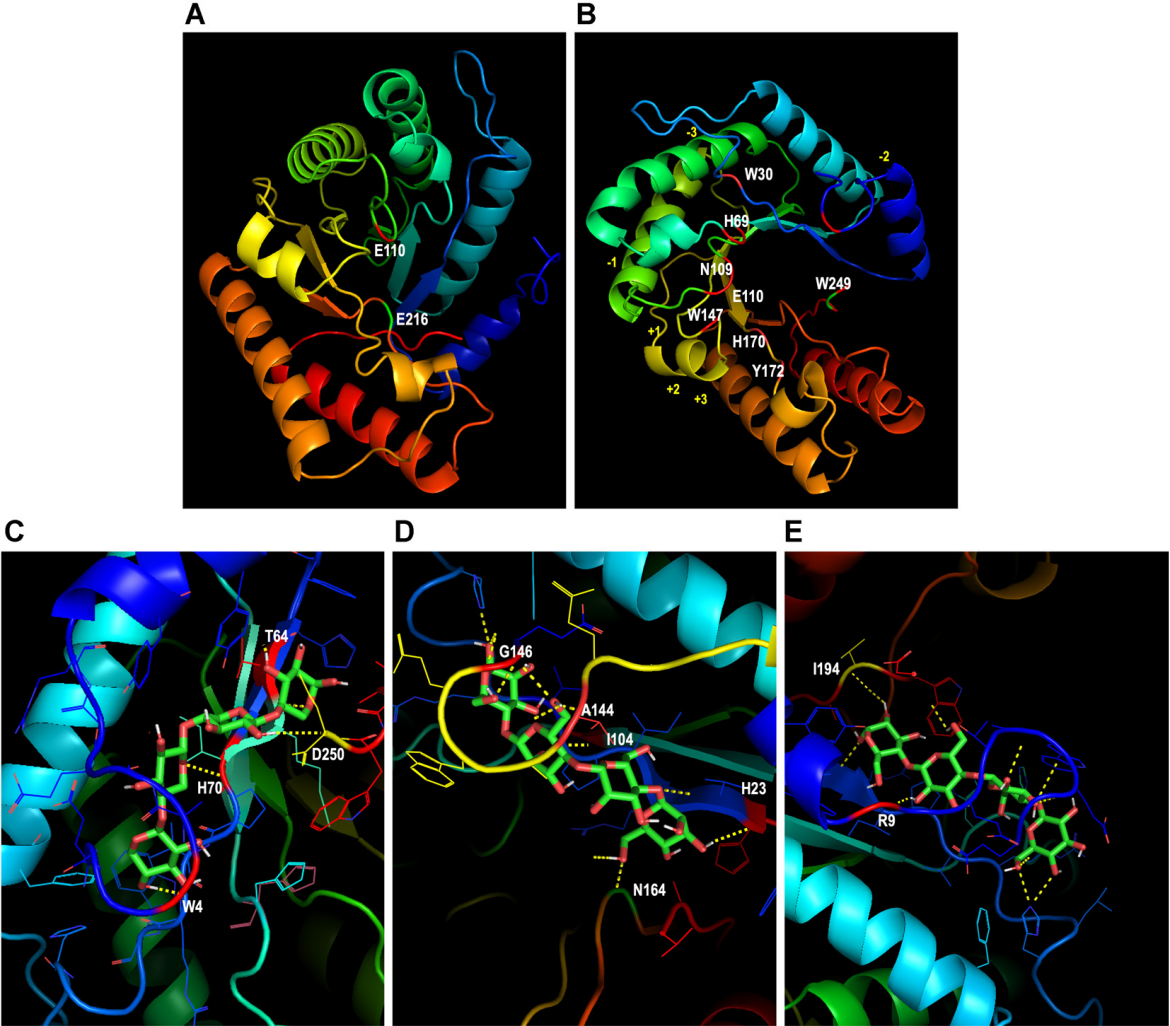
(A) The predicted 3D structure of *Pc*GH5_CD enzyme indicating the two amino acids residues (Glu110 and Glu216) at the catalytic site. (B) Amino acid residues (Trp30, His69, Asn109, Glu110, Trp147, His170, Tyr172, and Trp249) within the binding subsites for cellulase activity. (C, D, and E) Amino acid residues on the surface of the binding subsites of the *Pc*GH5_CD enzyme structure that are expected to be important for xylanase, mannanase, and β-1,3-1,4-glucanase activities, respectively. The xylotetraose (X4), mannotetraose (M4), and 1,3-1,4-β-glucotetraose (G4) represent sugar ligands for endo-xylanase, endo-mannanase, and endo-β-glucanase, respectively, which interact with *Pc*GH5_CD at subsites −2 to +2. The distances between interacting amino acid residues within these subsites and the ligands are presented by dotted lines. The 3D structure model was built based on the 4U3A reference protein model using SWISS-MODEL (https://swissmodel.expasy.org/). Ligands were obtained from PubChem (https://pubchem.ncbi.nlm.nih.gov/), while ligand-macromolecule interactions were analyzed with PyRx software to predict binding interactions [17]. Structural visualization and analysis were performed using PyMOL (https://pymol.org/).

To confirm the substrate specificity of the *Pc*GH5_CD enzyme and to obtain an in-depth understanding of the substrate-binding properties, structure modeling study on the binding of this enzyme was built by SWISS-MODEL using the crystal structure (PDB ID: 4U3A) of bifunctional endo-cellulase/endo-xylanase *Ct*Cel5E from *C. thermocellum* ATCC 27405 as the reference protein. This protein showed high sequence identity with the *Pc*GH5_CD enzyme and the 3D structure and binding subsites of this enzyme has already been reported [16]. Fig. 5A shows the two amino acids residues (Glu110 and Glu216) at the catalytic site of the *Pc*GH5_CD enzyme. Eight amino acid residues (Trp30, His69, Asn109, Glu110, Trp147, His170, Tyr172, and Trp249) within the binding subsites for endo-cellulase of the *Pc*GH5_CD is shown in Fig. 5B. Amino acid residues on the surface of the binding subsites of the *Pc*GH5_CD enzyme structure that located outside the active site of endo-cellulase, and are expected to be important for enzyme activities were detected; for endo-xylanase, such as Trp4, Thr64, His70, and Asp250 (Fig. 5C), endo-mannanase, such as His23, Ile104, Ala144, Gly146, and Asn164 (Fig. 5D), and endo-β-1,3-1,4-glucanase, such as Arg9 and Ile194 (Fig. 5E).

Table 2 shows the molecular dockings of molecular ligands into the active site of the *Pc*GH5_CD enzyme within the four binding subsites between −2 and + 2 (sequence from the reducing end) interacted with each ligand (X4, M4 or G4). For endo-xylanase, the hydrophobic stacking interaction of Xyl1 with aromatic residue of Trp4 was displayed on subsite −2 at distance of 2.6 Å. Whereas His70, Asp250, and Thr64 interacted with Xyl2, Xyl3 and Xyl4 at distances of 4.9, 3.4 and 2.0 Å, respectively. For endo-mannanase, His23 and Asn164 interacted with Man1 at distances of 2.4 and 3.5 Å, respectively. Whereas Ile104 and Ala144 interacted with Man3 at distances of 2.9 and 3.5 Å, respectively. Moreover, Gly146 interacted with Man4 at distance of 4.1 Å. For endo-1,3-1,4-β-glucanase, Arg9 interacted with Glu3 at distance of 3.5 Å, while Ile194 interacted with Glu4 at distance of 4.7 Å.

### Expression and Purification of Recombinants *Pc*GH5_CD and *Pc*GH5

**Fig. 6:**
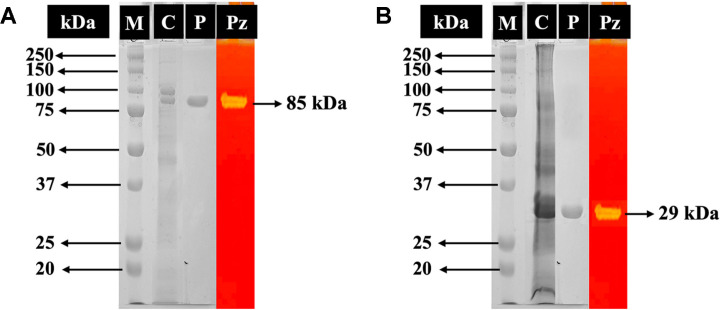
SDS-PAGE and cellulase zymogram analysis of purified recombinant proteins. (**A**) purified full-length *Pc*GH5 and (**B**) purified *Pc*GH5_CD catalytic domain. Lane M, protein marker; lane C, crude enzyme; lane P, purified fulllength *Pc*GH5 (85 kDa) or purified *Pc*GH5_CD catalytic domain (29 kDa); and lane Pz, zymogram analysis of purified fulllength *Pc*GH5 or purified *Pc*GH5_CD catalytic domain.

In this work, the two genes, namely *pcgh5* (full sequence) and *pcgh5_CD* (only GH5 catalytic domain) were ligated into the pET-28a vector, yielding pET28*Pc*GH5 and pET28*Pc*GH5_CD, respectively, which were introduced into *E. coli* DH5α. Recombinants full-length *Pc*GH5 and *Pc*GH5_CD catalytic domain were successfully expressed in *E. coli* BL21 (DE3). Both proteins contain a fusion protein with an *N*-terminal histidine tag that were purified with a HisTrap HP column. After purification, *Pc*GH5 and *Pc*GH5_CD showed a single band on SDS-PAGE and cellulase-zymogram with molecular masses of approximately 85 kDa and 29 kDa, respectively (Fig. 6A and 6B), which was consistent with the calculated values. These purified proteins will be used for further biochemical studies.

### Substrate Specificity of the *Pc*GH5_CD Enzyme

According to the CAZy enzyme classification (http://www.cazy.org/GH5.html), although most GH5 members display endo-cellulase activity, such as the endo-cellulase Cel5A from *T. aurantiacus* [22], a few other enzymes have also been reported, *e.g.* endo-xylanase PfXyn5 from *Penicillium funiculosum* [26], endo-mannanase AtMan5-1 from *Arabidopsis thaliana* [27], endo-β-1,3-1,4-glucanase PpBglu5A from *P. polymyxa* KF-1 [28], endo-β (1,3)-specific endo-glucanase GH5 from the human gut bacterium *Segatella copri* [5], β-glucosidase GH5BG from *Oryza sativa* [29], β-xylosidase T81Xyl5_22A from *Thermogemmatispora* sp. T81 [30], β-mannosidase *Ac*Man5 from the filamentous fungus *Absidia corymbifera* D1 [31], bifunctional endo-cellulase/endo-xylanase *Ct*Cel5E from *C. thermocellum* [16], bifunctional endo-cellulase/endo-mannanase *Ba*Man5A from *B. agaradhaerens* [23], multifunctional endo-cellulase/endo-mannanase/endo-β-glucanase from *Thermotoga maritima* [32] and multifunctional endo-cellulase/endo-mannanase/endo-β-glucanase Man5A from *C. polysaccharolyticus* [7].

Until now, only a few multifunctional enzymes of GH5 members such as two endo-cellulase/endo-mannanase/endo-β-glucanase from *T. maritima* [32] and *C. polysaccharolyticus*has have been reported [7]. However, information on these enzymes is limited. Therefore, it would be interesting to study enzymes in the GH5 family that have multifunctional enzyme properties. To study the substrate specificity, the *Pc*GH5_CD catalytic domain was examined against β-1,4 glycosidic linkages of polysaccharide substrates such as cellulose, xylan and mannan (KGM); β-1,4-1,6 glycosidic linkages of polysaccharide substrate such as mannan (LBG); β-1,3-1,4 glycosidic linkages of polysaccharide substrates such as barley β-glucan; α-1,4 and/or α-1,6 glycosidic linkages of polysaccharide substrates such as starch and pectin; β-1,4 glycosidic linkages of disaccharide substrates, as well as cellulose, xylan, and mannan contained in agricultural residues.

As shown in Table 3, the *Pc*GH5_CD catalytic domain effectively hydrolysed CMC (26.80 U mg^-1^ protein) and Avicel (15.58 U mg^-1^ protein). For xylan, the *Pc*GH5_CD enzyme hydrolysed BWX (16.21 U mg^-1^ protein) better than OSX (3.33 U mg^-1^ protein). For mannan, *Pc*GH5_CD preferred to hydrolyse KGM (18.33 U mg^-1^ protein) rather than LBG (3.33 U mg^-1^ protein). Moreover, *Pc*GH5_CD was active on barley β-glucan (4.54 U mg^-1^ protein). However, the *Pc*GH5_CD enzyme could not hydrolyse disaccharides (cellobiose, xylobiose and mannobiose), starch and pectin. Interestingly, the *Pc*GH5_CD enzyme, which contains only the catalytic domain, was able to hydrolyse cellulose, xylan, mannan and β-glucan, which are rarely found in the GH5 members (Fig. 2).

Without non-catalytic domains (CBM11-Fn3-CBM3), the *Pc*GH5_CD catalytic domain had higher activity on CMC than Avicel (Table 3). CMC is a short, single-chain derivative of cellulose that has some hydroxyl groups of its backbone replaced with carboxymethyl groups to make it more soluble [33]. Consequently, CMC is highly soluble and an easy substrate for cellulases to hydrolyse, whereas Avicel is a microcrystalline cellulose with high crystallinity. Thus, *Pc*GH5_CD was more effective against CMC than Avicel. Similarly, almost all GH5 endo-cellulases preferred to hydrolyse CMC rather than Avicel [34].

The hydrolysis of *Pc*GH5_CD enzyme toward BWX, the low-branching xylan, was more effective than that of the high-branching xylan, OSX (Table 3), where BWX is composed of 94% xylose and 6% other substituents, whereas OSX is composed of 52% xylose and 48% other substituents [35]. The substrate binding site of an enzyme is often hindered by substituted side groups of the substrate. Phakeenuya *et al*. [1] reported that the frequency of side chain substitution has a significant effect on the enzyme’s activity.

The *Pc*GH5_CD enzyme preferred to hydrolyse KGM rather than LBG. KGM is a β-D-1,4-linked glucopyranose and β-D-mannopyranose heteropolymer with a molar ratio of 1.0:1.6 in a linear chain. In addition, every 9–19th sugar unit in KGM contains an acetyl side group, which facilitate the solubility of KGM [36], whereas LBG is a galactomannan, which is made up of a backbone consisting of β-D-1,4-linked mannopyranose with branching of 1,6-linked α-D-galactopyranose spaced approximately every 3.5 mannose units [37], which probably obstructs enzyme function. Moreover, the nature of the GH5 members are endo-cellulases (Fig. 2) that prefers to hydrolyse the β-1,4 glycosidic linkage between β-D-1,4-linked glucose-glucose molecules. Thereby, this also may be the reason for the efficient hydrolysis of the *Pc*GH5_CD enzyme toward the β-1,4 glycosidic linkage between glucose-mannose molecules of KGM compared with galactose-mannose of LBG (Table 3).

Barley β-glucan is a linear chain of the β-glucopyranosyl unit, of which approximately 70% is joined by β-1,4 glycosidic linkage and approximately 30% by β-1,3 glycosidic linkage [38]. Unexpectedly, the *Pc*GH5_CD enzyme could hydrolyse barley β-glucan, which is rarely found in GH5 members. Compared with other substrates, *Pc*GH5_CD may be suitable for substrates with only β-1,4 glycosidic linkages, resulting in low enzyme activity on this substrate. Endo-β-1,3-1,4-glucanase of GH5 from *P. polymyxa* KF-1 has been reported. This enzyme showed high activity on barley β-glucan and lichenan [28]. Recently, endo-β (1,3)-specific endo-glucanase belonging to GH5 from the human gut bacterium *Segatella copri* has been reported [5].

Although some GH5 members have been shown to react with β-1,4-linked glycosidic disaccharides, such as β-glucosidase from *Oryza sativa* [29], β-xylosidase from *Thermogemmatispora* sp. T81 [30], and β-mannosidase from the filamentous fungus *Absidia corymbifera* D1 [31]; however, the activity of these enzymes was not detected in *Pc*GH5_CD enzyme. On the other hand, all members of GH5 were unable to hydrolyse the α-1,4 and/or α-1,6 glycosidic linkages of polysaccharide substrates, including *Pc*GH5_CD from *P. curdlanolyticus* strain B-6.

The *Pc*GH5_CD enzyme from *P. curdlanolyticus* B-6 could hydrolyse β-1,4 glycosidic linkages of polysaccharides and related structures (β-1,4-1,6 and β-1,3-1,4 glycosidic linkages of polysaccharides), but not for β-1,4 glycosidic linkages of disaccharides and α-1,4 and/or α-1,6 glycosidic linkages of polysaccharides. These results revealed that the *Pc*GH5_CD enzyme exhibits multifunctional enzyme activities against various polysaccharide substrates, including cellulose, xylan, mannan and β-glucan. Thus, the *Pc*GH5_CD enzyme exhibits unique properties of multifunctional endo-cellulase/endo-xylanase/endo-mannanase/endo-β-glucanase activities that have never been reported before.

Although cellulose, xylan, mannan and β-glucan are the main polysaccharide components in agricultural residues, the *Pc*GH5_CD enzyme could not hydrolyse these polysaccharides contained in sugarcane bagasse and coconut meal (Table 3). This may be due to the complexity of these polysaccharides in the structures of agricultural residues. Kumar *et al*. [39] reported on the structural complexity of the agricultural residue, in which xylan is densely packed with lignin layers through ether-ester linkages and interacts with cellulose microfibrils through hydrogen bonds, protecting polysaccharide substrates from enzymatic hydrolysis.

### Substrate Specificity of Full-Length *Pc*GH5 Enzyme on Polysaccharides

To study the effect of the non-catalytic domains (CBM11-Fn3-CBM3) linked to the *Pc*GH5_CD catalytic domain, the activities of full-length *Pc*GH5 were tested against various polysaccharide substrates. Compared with the *Pc*GH5_CD catalytic domain, for cellulose substrates, the enzyme activity of full-length *Pc*GH5 toward CMC (33.79 U mg^-1^ protein) was slightly increased by 1.26-fold, while that of Avicel (28.04 U mg^-1^ protein) was increased by 1.80-fold (Table 3). For xylan substrates, the activity of the *Pc*GH5 enzyme toward BWX (30.74 U mg^-1^ protein) and OSX (10.09 U mg^-1^ protein) were improved 1.90-fold and 3.03-fold, respectively. For mannan substrates, the enzyme activity on KGM (29.26 U mg^-1^ protein) and LBG (17.37 U mg^-1^ protein) increased by 1.60-fold and 5.22-fold, respectively. Furthermore, *Pc*GH5 enzyme activity on barley β-glucan (49.59 U mg^-1^ protein) significantly increased the enzyme activity (10.04-fold) (Table 3). These results indicate that the three non-catalytic domains linked to the GH5 catalytic domain of *P. curdlanolyticus* B-6 have a significant effect on improving the catalytic activity toward crystalline cellulose, highly branched polysaccharides, and β-1,4-1,6 and β-1,3-1,4 glycosidic linkages of polysaccharides. We plan to study the role of each non-catalytic domain on the function of full-length *Pc*GH5 in the near future.

It has been reported that CBM3s show binding affinity for cellulose, xylan and mannan [40] and can promote the activity of the catalytic domain in glycoside hydrolase activities toward polysaccharides and complex substrates present in agricultural residue [41, 42], whereas the presence of Fn3 can modify the cellulose surface to increase cellulase activity against insoluble cellulose [11]. Alternatively, the presence of CBM11 could improve the affinity of the catalytic domain for 1,3-1,4-β-glucan [43]. Accordingly, when these non-catalytic domains (CBM11-Fn3-CBM3) associate with the GH5 catalytic domain of *P. curdlanolyticus* strain B-6 (Fig. 1A), it may facilitate substrate recognition and orient the active site of the enzyme in the correct direction to improve the enzyme’s activity on cellulose, xylan, mannan and β-glucan substrates. On the other hand, enzyme activities toward sugarcane bagasse and coconut meal were observed in full-length *Pc*GH5 only, not in the catalytic domain (*Pc*GH5_CD) (Table 3). As a result, these non-catalytic domains linked to the GH5 catalytic domain of the strain B-6 should be key factors in improving the enzyme activities toward various β-1,4 glycosidic linkages of polysaccharide substrates, especially hemicellulose (xylan and mannan) and cellulose contained in agricultural residues.

Previous studies demonstrated that some non-catalytic domains linked to the catalytic domains of glycoside hydrolases are involved in the specific activity of the enzymes toward insoluble and/or complex structure substrates. For example, the presence of CBM36 was found to be necessary for the endo-xylanase property of the multifunctional arabinofuranosidase/endo-xylanase/β-xylosidase PcAxy43B enzyme from *P. curdlanolyticus* B-6 [10]. Ye *et al*. [44] reported that when a new CBM with sequence similarity to CBM29-2 from *Meiothermus taiwanensis* WR-220 was combined with the endo-cellulase catalytic domain of Cel5A of *T. maritima*, the substrate affinity of *Tm*Cel5A increased to make a robust cellulase. Deletion of the two CBM16s from Man5A of *Thermoanaerobacterium polysaccharolyticum* resulted in a significant loss of both endo-mannanase and endo-cellulase (CMCase) activities [45]. Similarly, deletion of the two CBM16s from Man5B of *C. polysaccharolyticus* resulted in a significant loss of endo-mannanase activity toward LBG, guar gum and β-mannan [7]. Alternatively, the two CBM22s of the full-length bifunctional endo-xylanase/endo-β-1,3-1,4-glucanase Xyn10B from *C. stercorarium* are important for β-1,3-1,4-glucanase activity toward barley β-glucan [46]. Moreover, the replacement of CBM11 in the endo-cellulase *Ct*Cel5E from *C. thermocellum* with low xylanase activity by CBM6 from *C. stercorarium* significantly increased the xylanase specificity activity of *Ct*Cel5E toward oat spelt xylan [47].

### Mode of Action of the *Pc*GH5_CD Enzyme on Polysaccharides

**Fig. 7:**
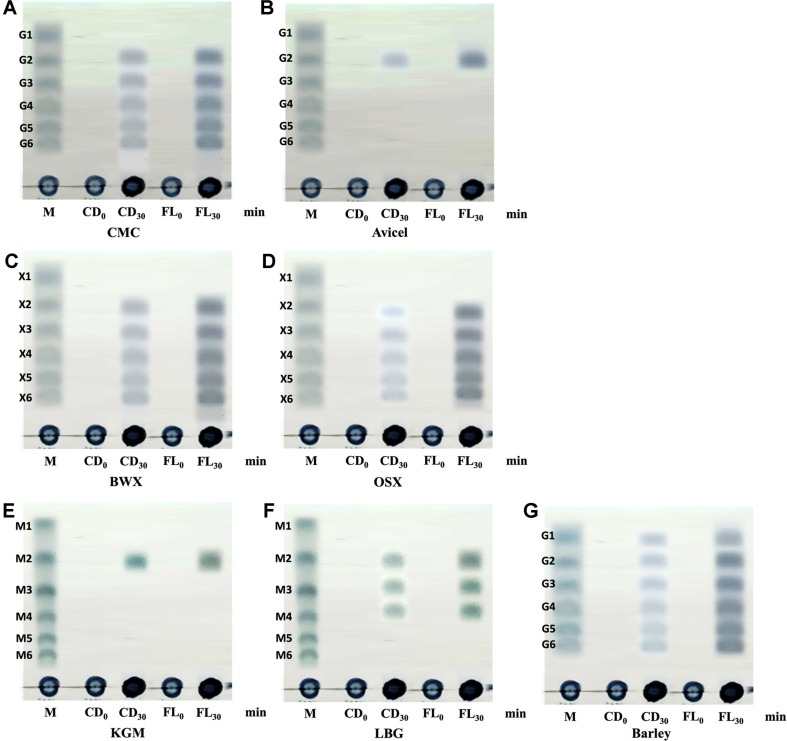
TLC analysis of hydrolysis products released from polysaccharides by purified *Pc*GH5_CD (CD) and full-length *Pc*GH5 (FL). Polysaccharides tested include CMC (**A**), Avicel (**B**), BWX (**C**), OSX (**D**), KGM (**E**), LBG (**F**), and barley β-glucan (**G**). Lane M represents size markers: glucose and cellooligosaccharides (G1–G6), xylose and xylooligosaccharides (X1–X6), and mannose and manno-oligosaccharides (M1–M6). Lane CD_0_ or FL_0_ represents the control, and lane CD_30_ or FL_30_ represents the reaction at 30 min. Each polysaccharide (1%, w/v) was incubated with *Pc*GH5_CD or *Pc*GH5 (1 μM) in sodium acetate buffer (pH 6.0) at 50°C. For inactive enzyme control, *Pc*GH5_CD or *Pc*GH5 was boiled for 15 min before use.

Due to the ability of *Pc*GH5_CD to hydrolyse various β-1,4 glycosidic linkages and the related structure of polysaccharides (Table 3), the product patterns of the *Pc*GH5_CD enzyme on these substrates were analysed. Products released from CMC and Avicel had different patterns. The CMC products were a series of cello-oligosaccharides G6, G5, G4, G3 and G2 (Fig. 7A). This result indicates the endo-action of the *Pc*GH5_CD enzyme on the CMC substrate. On the other hand, for Avicel, cellobiose (G2) was the only product obtained (Fig. 7B). Avicel (microcrystalline cellulose) is a highly crystalline cellulose in which the numerous cellulose chains in the Avicel structure are held tightly together by hydrogen bonds. Moreover, the substrate binding groove of the GH5 enzyme is narrow and long [22]. Therefore, it did not allow the *Pc*GH5_CD enzyme to attack the inner regions of the Avicel structure. However, this endo-acting enzyme attempts to produce sugar(s) from the Avicel chain ends (likely for the growth of *P. curdlanolyticus* B-6 cells), which have a loose structure, and released cellobiose as a single product. This phenomenon is observed in some endo-cellulases that hydrolyse crystalline cellulose. For example, cellobiose was the main product of Avicel hydrolysis by an endo-cellulase from *Bacillus circulans* F-2 [48], the main product released from Avicel by the brown-rot basidiomycete *Fomitopsis palustris* endo-cellulase was cellobiose [49], a processive endo-cellulase from the brown rot basidiomycete *Gloeophyllum trabeum* hydrolysed Avicel to cellobiose as the major product [50], and an endo-cellulase from *Trichoderma reesei* CDU-11 produced cellobiose as the main product from Avicel [51].

For xylan substrates, the *Pc*GH5_CD enzyme hydrolysed BWX and OSX to a series of xylo-oligosaccharides. However, the hydrolysis product patterns of BWX and OSX were clearly different. X6, X5, X4, X3 and X2 were products released from BWX, whereas the products from OSX were a series of oligosaccharides, which were not consistent with the size of standard sugars (Fig. 7C and 7D), which might be due to the effect of its substitute groups. For mannan substrates, the *Pc*GH5_CD enzyme degraded KGM and released only the dimeric product, which may be mannose-mannose or mannose-glucose (Fig. 7E). For LBG, a series of tetrameric, trimeric and dimeric mannose-mannose and/or mannose-galactose molecules were detected (Fig. 7F). These results indicate that the hydrolysis products of the enzyme depend on the type of substrate. Phakeenuya *et al*. [1] reported that the type of substrate affected the function of the enzyme. Alternatively, the *Pc*GH5_CD enzyme could hydrolyse barley β-glucan and generated a series of gluco-oligosaccharides (Fig. 7G).

As a result of the mode of action of the full-length *Pc*GH5_CD on polysaccharides, the *Pc*GH5_CD enzyme produced various types of oligosaccharides that can be used in a variety of food products. For example, cello-oligosaccharides have prebiotic properties and can stimulate the growth of many probiotics, such as *Lactobacillus* and *Bifidobacterium* [52]. Xylo-oligosaccharides have acceptable stability and sensory properties, which can be used as prebiotics and low-caloric food sweeteners without toxicity or adverse effects on human health [53]. Manno-oligosaccharides are used as prebiotics due to their ability to promote the growth of *Lactobacilli* and *Bifidobacteria*, biofilm formation and the production of beneficial short-chain fatty acids [54], while gluco-oligosaccharides can be used as prebiotics to improve the health of humans and animals [5].

### Mode of Action of Full-length *Pc*GH5 Enzyme on Polysaccharides

The mode of action of the full-length *Pc*GH5 enzyme towards all polysaccharide substrates was similar to that of the *Pc*GH5_CD enzyme. However, the amounts of products produced by the full-length enzyme were higher than that of the *Pc*GH5_CD enzyme for all polysaccharides tested (Fig. 7), which may be due to the role of non-catalytic domains linked to the *Pc*GH5_CD catalytic domain.

### Hydrolysis of Untreated Agricultural Residues by Full-length *Pc*GH5 Enzyme

Sugarcane is grown in large quantities in tropical and subtropical countries. In 2020, approximately 1.9 billion tons of sugarcane were produced worldwide (https://en.wikipedia.org › wiki › Sugarcane). Sugarcane bagasse is the solid by-product of sugarcane juice extraction and constitutes approximately 30% of the material remaining after extraction. Sugarcane bagasse contains 51.3% cellulose, 33.5% xylan and 8.20% lignin [55]. Alternatively, coconut is an important plant in several Asian and South American countries, with an annual production of more than 58 million tons around the world, whereas coconut meal is a by-product of coconut milk production, which amounts to approximately 3,600 tons per year. Coconut meal is composed of 55.03% mannan, 8.83% cellulose and 25.59%fat [56]. Sugarcane bagasse and coconut meal occur as by-products in large quantities in Thailand. Therefore, we selected sugarcane bagasse (representative of natural xylan and cellulose) and coconut meal (representative of natural mannan and cellulose) to test the ability of the full-length *Pc*GH5 enzyme to hydrolyse these residues without pretreatment (only cut into small pieces).

Surprisingly, although the *Pc*GH5_CD catalytic enzyme was unable to hydrolyse agriculture residues, the full-length *Pc*GH5 enzyme showed hydrolytic activity on sugarcane bagasse (20.58 U mg^-1^ protein) and coconut meal (12.98 U mg^-1^ protein) (Table 3). The *Pc*GH5 enzyme released reducing sugars from sugarcane bagasse better than coconut meal, which may be due to the higher content of xylan and cellulose in sugarcane bagasse compared with that of mannan and cellulose in coconut meal. Moreover, coconut meal is high in fat (25.6%), which may interfere with the action of the *Pc*GH5 enzyme on polysaccharide substrates. The *Pc*GH5 enzyme can hydrolyse polysaccharides that exist in the complex structure of agricultural residues. These results indicate the cooperative action between the GH5 catalytic domain and the three non-catalytic domains (CBM11-Fn3-CBM3) from *P. curdlanolyticus* strain B-6 to promote the enzyme activities of the catalytic domain toward naturally insoluble substrates present in agricultural residues, which has never before been reported in GH5 members. Similarly, Limsakul *et al*. [10] reported that the presence of CBM36 is required for the multifunctional arabinofuranosidase/endo-xylanase/β-xylosidase PcAxy43B enzyme from *P. curdlanolyticus* B-6 to hydrolyse β-1,4 glycosidic linkages of polysaccharide (xylan) contained in corn hull. Moreover, Pasari *et al*. [57] reported on the importance of X2-CBM3 modules in agricultural residue (wheat straw) hydrolysis and provided insights into how CBM engineering can be applied to improve cellulosic enzyme binding affinity and hydrolysis potential.

**Fig. 8:**
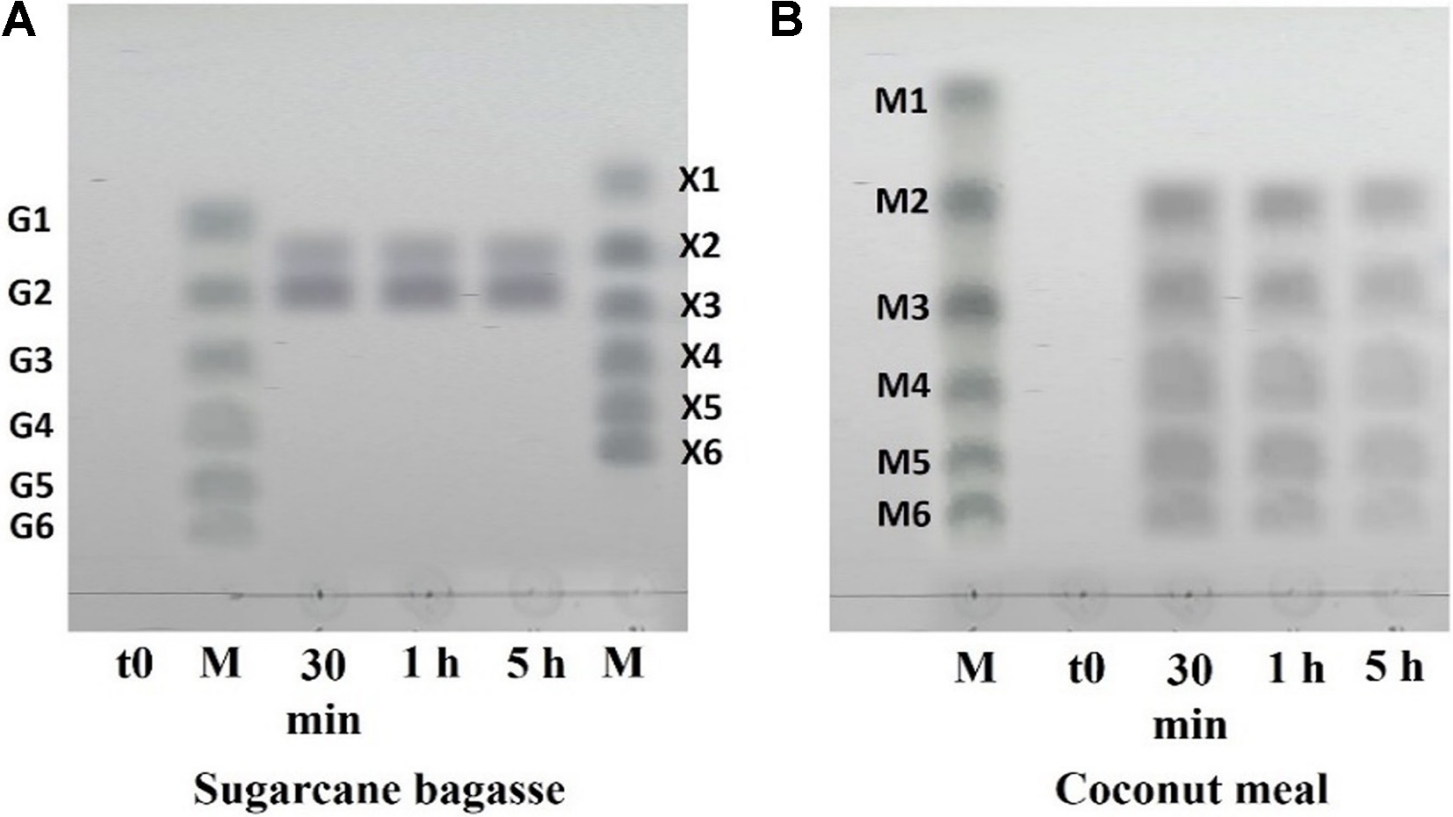
TLC profiles of hydrolysis products generated from agricultural residues by purified full-length *Pc*GH5. (**A**) Sugarcane bagasse. (**B**) Coconut meal. Lane t0 represents the control. Lane M represents size markers: glucose and cellooligosaccharides (G1–G6), xylose and xylooligosaccharides (X1–X6), and mannose and manno-oligosaccharides (M1–M6). Lanes 30 min, 1 h, and 5 h represent reactions at the respective time points. Reactions were performed by incubating each substrate (1%, w/v) with 5 μM *Pc*GH5 in sodium acetate buffer (pH 6.0) at 50°C for 30 min, 1 h, and 5 h. For inactive enzyme control, *Pc*GH5 was boiled for 15 min before use.

TLC chromatograms demonstrated that the full-length *Pc*GH5 enzyme generated X2 and G2 as major products from sugarcane bagasse (Fig. 8A). This result may be due to the non-catalytic domains (CBM11-Fn3-CBM3) being important for the *Pc*GH5_CD catalytic domain to deconstruct the complex structure of agricultural residues, resulting in a structural change that allows *Pc*GH5 xylanase to hydrolyse xylan located on the surface of sugarcane bagasse to produce X2. Thereafter, the non-catalytic domains may modify the cellulose surface by Fn3 [11] and may disrupt the hydrogen bond network of crystalline cellulose by CBM3 [58], resulting in swelling and loosening of the cellulose-xylan matrix and crystalline structure. These effects result in an increase in amorphous regions and cellulose surface area and improve the accessibility of cellulase to cellulose, thus producing G2.

The full-length *Pc*GH5 enzyme was able to release a series of manno-oligosaccharides M6, M5, M4, M3 and M2 from coconut meal (Fig. 8B), probably due to the interaction between the endo-mannanase of the catalytic domain and CBM11, where it was reported that CBM11 enhanced the affinity of the catalytic domain to 1,3-1,4-β-glucan [43], but no cellulosic products were detected. These results indicate that the cellulose present in coconut meal is too strong for *Pc*GH5 to hydrolyse, which might be because the cellulose in coconut meal is coated with a high amount of fat [56], making the cellulose resistant to the action of endo-cellulase. The advantage of this result is that only manno-oligosaccharides from coconut meal were obtained.

## Conclusion

This work presents a novel multifunctional endo-cellulase/endo-xylanase/endo-mannanase/endo-β-glucanase GH5 enzyme from *P. curdlanolyticus* B-6, and the role of its non-catalytic domains. This enzyme could hydrolyse the β-1,4 glycosidic linkages of polysaccharides present in agricultural residues into oligosaccharides in one step without any pretreatment. This environment-friendly enzymatic hydrolysis has received increasing attention in recent years. Therefore, *Pc*GH5 is a good candidate to produce oligosaccharides from agricultural residues for use as prebiotics in various food products.

## Figures and Tables

**Table 1:** Comparison of conserved amino acid residues in the active site including the catalytic site and substrate-binding subsites of endo-cellulase from *T. aurantiacus* [22] with other characterized GH5 enzymes with different functions from the catalytic domain (*Pc*GH5_CD) enzyme of *P. curdlanolyticus* strain B-6 (this work), bifunctional endo-cellulase/endo-xylanase from *C. thermocellum* [16], bifunctional endo-cellulase/ endo-mannanase from *B. agaradhaerens* [23], multifunctional endo-cellulase/endo-mannanase/endo-β-glucanase from *C. polysaccharolyticus* [7], and endo-β-1,3-1,4-glucanase from *P. polymyxa* KF-1 [25].

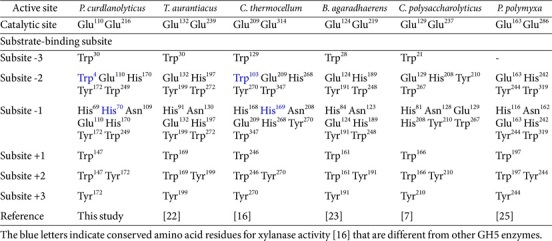

**Table 2:** Summary of molecular dockings of X4, M4, and G4 into each amino acid residue located within the four binding subsites of *Pc*GH5_CD.

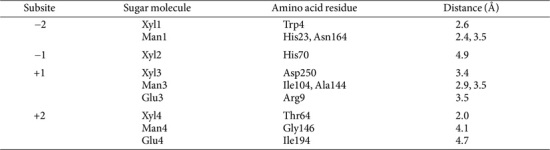

**Table 3:** Substrate specificity of purified *Pc*GH5_CD catalytic domain enzyme and the full-length *Pc*GH5 enzyme from *P. curdlanolyticus* strain B-6 toward various substrates.